# Structural Properties Characterized by the Film Thickness and Annealing Temperature for La_2_O_3_ Films Grown by Atomic Layer Deposition

**DOI:** 10.1186/s11671-017-2018-8

**Published:** 2017-03-29

**Authors:** Xing Wang, Hongxia Liu, Lu Zhao, Chenxi Fei, Xingyao Feng, Shupeng Chen, Yongte Wang

**Affiliations:** Key Laboratory for Wide-Band Gap Semiconductor Materials and Devices of Education, School of Microelectronics, Xidian University, Xi’an, 710071 China

**Keywords:** La_2_O_3_, ALD, Crystallization, Diffusion, Bandgap, Refractive index

## Abstract

La_2_O_3_ films were grown on Si substrates by atomic layer deposition technique with different thickness. Crystallization characteristics of the La_2_O_3_ films were analyzed by grazing incidence X-ray diffraction after post-deposition rapid thermal annealing treatments at several annealing temperatures. It was found that the crystallization behaviors of the La_2_O_3_ films are affected by the film thickness and annealing temperatures as a relationship with the diffusion of Si substrate. Compared with the amorphous La_2_O_3_ films, the crystallized films were observed to be more unstable due to the hygroscopicity of La_2_O_3_. Besides, the impacts of crystallization characteristics on the bandgap and refractive index of the La_2_O_3_ films were also investigated by X-ray photoelectron spectroscopy and spectroscopic ellipsometry, respectively.

## Background

During the past decades, lanthanum oxide (La_2_O_3_) has raised great research interests due to its remarkable chemical, thermal, optical, and electrical properties [1–3]. On the one hand, featuring with high dielectric constant (approximately 27) and large band offsets with silicon (over 2 eV), La_2_O_3_ is one among the most promising high-k dielectric materials to replace SiO_2_ and Si_3_N_4_ in advanced metal-oxide gate stack in semiconductor devices [4]. Up to now, benefiting from the approach of surface passivation prior to oxide deposition, high-quality ceria/lanthana gate stack suitable for high-k integration in a gate-last process has been accomplished [5]. On the other hand, La_2_O_3_ is usually used as a kind of effective dopant in thermionic emitters [6], ferroelectric ceramics [7], and oxide catalysts [8], in order to improve properties such as emission capability, effective dielectric constant, and catalytic activity. Besides, La_2_O_3_ thin films have also received increasing attentions for the various applications in glass ceramic [9], gas sensor [10], supercapacitor [11], etc.

La_2_O_3_ thin films have been prepared by various physical and chemical deposition methods, such as electron beam evaporation [12], vacuum evaporation [13], chemical vapor deposition [14], atomic layer deposition (ALD) [15], and molecular beam epitaxy [16]. Among the deposition methods mentioned above, due to the nature of the self-limited reaction, ALD has been considered as one of the most promising deposition techniques to produce high quality La_2_O_3_ thin films with atomic scale thickness controllability, fine uniformity, and excellent conformality [17]. La_2_O_3_ thin films can be found in several crystalline phases, namely, hexagonal (*h*-La_2_O_3_), cubic (*c*-La_2_O_3_), amorphous (*a*-La_2_O_3_), or a mixture of the phases depending on the film deposition method and post-deposition heat treatment [18]. It is well known that the structural properties of La_2_O_3_ thin film are determined, to a large extent, by its crystallization and microscopic morphology [19]. Therefore, the study of the crystallization and structure of La_2_O_3_ thin film is of great significance for the compatibility of the film application into advanced electronic devices. In this article, the structural properties of La_2_O_3_ thin films prepared by ALD technique were investigated by means of a variety of measurements. Attentions were focused on the crystallization conditions of La_2_O_3_ film and the structural properties characterized by the crystalline states.

## Methods

La_2_O_3_ films were deposited on p-type Si (100) wafers in an atomic layer deposition reactor (Picosun R-150) using La(^i-^PrCp)^3^ as the La precursor while O_3_ was used as the oxidant. Prior to deposition of the films, native SiO_2_ was removed in a diluted HF solution (1:50). At the deposition temperature of 300 °C, a steady-state growth rate of ~0.85 Å/cycle is obtained by optimizing the process parameters (0.1 s La(^i-^PrCp)_3_ pulse/4 s purge with N_2_/0.3 s O_3_ pulse/10 s purge with N_2_). Ten and twenty nanometer La_2_O_3_ films were prepared by varying the number of ALD cycles. For both the 10 and 20 nm La_2_O_3_ films, post-deposition rapid thermal annealing (RTA) was carried out at 400, 600, and 800 °C for 60 s in vacuum ambient (~1 mbar). The ellipsometric spectra of La_2_O_3_ films were measured before and after annealing by spectroscopic ellipsometry (SE) system (J.A.Woollam Co. M2000U, Lincoln, NE, USA) over the wavelength range from 245 to 1000 nm. In order to address the evolution of the crystallographic structure, grazing incidence X-ray diffraction (GIXRD) measurements were carried out at an angle of incidence of 1° on both the as-grown and annealed La_2_O_3_ films. Cross-sectional high-resolution transmission electron microscopy (HRTEM) and energy-dispersive X-ray spectroscopy (EDX) line scan measurements were performed with [100] direction of the Si substrate to observe the microstructures and atomic compositions of the La_2_O_3_ films. X-ray photoelectron spectroscopy (XPS) analysis on a Theta 300 XPS system from Thermo Fisher was employed to investigate the bandgaps of the deposited films. After being exposed to air in clean room environment with a relative humidity of 50% for 48 h, GIXRD and HRTEM measurements were carried out on the as-grown and annealed La_2_O_3_ films again for further analysis.

## Results and Discussion

**Fig. 1:**
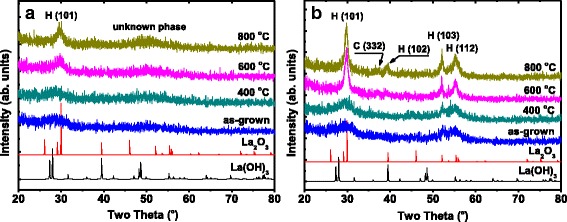
GIXRD diffractograms of as-grown and annealed La_2_O_3_ films deposited on Si substrate. **a** 10 and **b** 20 nm La_2_O_3_ films. Hexagonal La_2_O_3_ and hexagonal La(OH)_3_ patterns are added for comparison

Figure 1 illustrates the GIXRD analysis performed on the as-grown and annealed La_2_O_3_ films. The powder patterns of *h*-La_2_O_3_ [20] and *h*-La(OH)_3_ [21] are added for comparison. As the GIXRD measurements were carried out immediately after the deposition and annealing process, no peaks attributed to La(OH)_3_ exist in the GIXRD diffractograms. The 10 nm La_2_O_3_ film (as shown in Fig. 1a) shows no diffraction features before and after a 400 °C annealing treatment, suggesting an amorphous disordered structure of the film. After being annealed at 600 and 800 °C, only weak crystalline planes such as hexagonal (101) appear [22, 23], indicating the impossibility of converting the 10 nm La_2_O_3_ film into complete crystalline phase. The very small and broad peak around 50° in the diffractogram of the 10 nm La_2_O_3_ film annealed at 800 °C does not fit to the *h*-La_2_O_3_ or *h*-La(OH)_3_ patterns. We think it may be formed under the influence of several crystalline planes of *h*-La_2_O_3_ around 50°. However, for the 20 nm La_2_O_3_, the as-grown film already shows a small degree of crystallinity with a couple of peaks attributed to *h*-La_2_O_3_ (as shown in Fig. 1b). After being annealing treated, the intensities of the GIXRD peaks increase, which means the enhancement in the degree of crystallinity. After annealing at 600 °C, except for the weak cubic (332) plane [14], the film was mainly crystallized to hexagonal phase as the GIXRD diffractograms exhibit strong hexagonal planes such as (101), (102), (103), and (112). Besides, further increase in the annealing temperature up to 800 °C does not seem to significantly affect the GIXRD diffractograms of the film. That is, upon 600 °C, the increase in the annealing temperature does not enhance the crystallinity of the film. Consequently, when annealed upon 600 °C, an almost complete crystallization could be accomplished for the 20 nm La_2_O_3_ film.

**Fig. 2:**
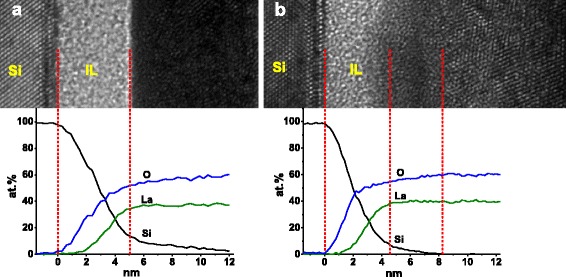
HRTEM images and EDX profiles near the interface for La_2_O_3_ films annealed at 600 °C. **a** 10 and **b** 20 nm La_2_O_3_ films

Additional structural information at the La_2_O_3_/Si interface after RTA treatment at 600 °C is provided by HRTEM-EDX analysis as shown in Fig. 2. For both 10 nm (Fig. 2a) and 20 nm (Fig. 2b) La_2_O_3_ films, the lanthanum and oxygen in-depth distributions in the EDX elemental ratio profiles show a parallel profile and the La/O ratio is close to 2:3 which meets well with the stoichiometry of La_2_O_3_. In the HRTEM images, an amorphous region between the Si substrate and the fabricated film, corresponding to an interfacial layer (IL) formed during the ALD growth and RTA process [24], could be found in both Fig. 2a, b. After the amorphous IL, it is possible to identify a region containing nanometer-sized crystals in the 10 nm La_2_O_3_ film, indicating the existence of an incomplete structural conversion (from amorphous to crystallographic structure) during the RTA treatment. However, the structure of the 20 nm La_2_O_3_ film is a little complicated. With the guidance of dotted lines, an amorphous region, a nanometer-sized crystal transition region, and a long-range ordered crystal region could be observed in the HRTEM image of Fig. 2b. The presence of long-range ordered crystals manifests, in accordance with the GIXRD results shown in Fig. 1, that RTA process upon 600 °C induces an almost complete crystallization of the 20 nm La_2_O_3_ film.

It is worth noting that upon the same annealing condition of at 600 °C for 60 s in vacuum ambient (~1 mbar), the 10 and 20 nm La_2_O_3_ films show different crystalline characteristics. We attribute this difference to the RTA-induced Si diffusion from the substrate into the La_2_O_3_ layer [25]. As we know, La_2_O_3_ exhibits the highest affinity for Si atoms among the rare-earth oxide films due to the so called “lanthanide contraction” property of rare-earth elements [26]. Even in the as-deposited La_2_O_3_ film grown by ALD method, substrate silicon atoms diffuse moderately and distribute in gradient from Si substrate to the upper layer, causing the presence of an IL about 1 nm [27, 28]. Besides, part of the as-deposited La_2_O_3_ film close to the IL could be considered as Si-riched and difficult to crystallize as Si rich help to prevent the formation of crystalline La_2_O_3_ precipitates [29]. Furthermore, post-deposition annealing causes extra silicon out diffusion and reaction with excess oxygen in the film. Consequently, in thin La_2_O_3_ film with the thickness of 10 nm or less, during the annealing process, the substrate Si atoms would diffuse deep easily to the upper layer before the film is crystallized. However, for the 20 nm as-deposited La_2_O_3_, since Si atoms distribute in gradient from Si substrate to the upper layer, a great part of the film relatively far away from Si substrate is pure. We think that this part of La_2_O_3_ film could be crystallized at appropriate post-deposition treatment such as RTA carried out at 600 and 800 °C for 60 s in vacuum ambient (~1 mbar) in this work. Crystallization of the film brings in an aggressive enhancement in the packing density and thermodynamic stability. Thus, to a certain extent, the diffusion of Si atoms from substrate into the upper layer would be restrained. As a result, the silicate layer of the 20 nm La_2_O_3_ film is slightly thinner than what could be observed in the 10 nm La_2_O_3_ film. Besides, in the 20 nm La_2_O_3_ film, only 3~4 nm La_2_O_3_ closed to the IL was converted into nanometer-sized crystals under the influence of Si diffusion during the annealing process. Complete crystallization of the as-grown film into the h-La_2_O_3_ phase is achieved in the region not affected by Si diffusion.

**Fig. 3:**
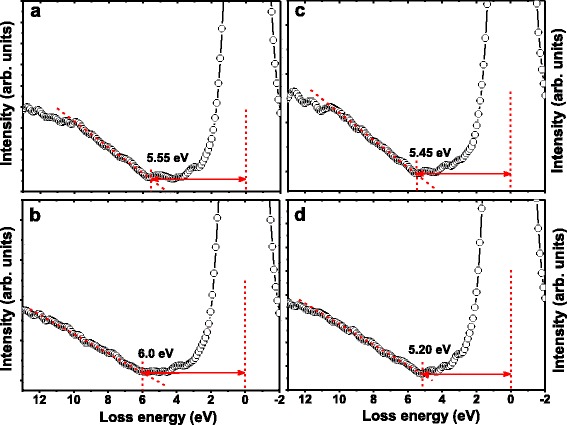
Bandgaps for the La_2_O_3_ films with different thickness and annealing temperatures. **a** as-grown 10 and **b** 10 nm La_2_O_3_ annealed at 600 °C, and **c** as-grown 20 and **d** 20 nm La_2_O_3_ annealed at 600 °C

The bandgaps of the as-grown and annealed (at 600 °C) La_2_O_3_ films were measured by examining the energy loss of the O 1s core levels as shown in Fig. 3. As we know, the bandgap equals the energy distance between the photoemission peak centroid and the onset of the features due to single particle excitations, and it is usually obtained from the inelastic energy loss features observed on the high binding energy side of the core level photoemission peaks [30]. The onset of O 1s loss spectrum was determined by linearly extrapolating the segment of maximum negative slope to the back ground level [31]. The bandgaps of the as-grown 10 and 20 nm La_2_O_3_ films are determined to be 5.55 and 5.45 eV, respectively. These values are in fairly good agreement with Ohmi et al. [32], who have reported a bandgap of 5.50 eV for non-crystallized La_2_O_3_ on Si substrate. The bandgap of the annealed 20 nm La_2_O_3_ film is determined to be 5.20 eV, which agrees well with the bandgap of 5.30 eV for crystallized La_2_O_3_ reported by Zhao et al. [33]. However, the diffusion of Si during the annealing process brings in large mounts of La-O-Si bonds for the 10 nm La_2_O_3_, leading to the increase of the inelastic energy loss during the transition from valence band to conduction band, which means the increment of bandgap [34]. As a result, the bandgap of the annealed 10 nm La_2_O_3_ is figured out as 6.0 eV, which is evidently larger than the bandgap of crystallized La_2_O_3_.

**Fig. 4:**
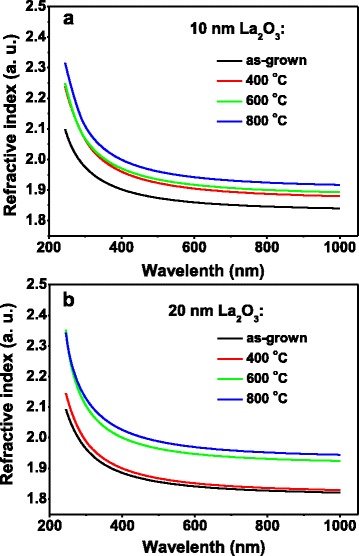
Annealing temperature dependence of refractive index for ALD-La_2_O_3_ with different thickness. **a** 10 nm La_2_O_3 _films and **b** 20 nm La_2_O_3 _films

Figure 4 illustrates the annealing temperature dependence of refractive indexes for the as-grown and annealed La_2_O_3_ films revealed by SE fitting. The refractive indexes of the La_2_O_3_ films were determined by fitting the ellipsometry data using the well-known Tauc-Lorentz dispersion mode, which was proposed by Jellison and Modine and has been successfully applied to a variety of amorphous and crystallized materials [35–37]. As revealed in Fig. 4, the refractive indexes of the as-grown La_2_O_3_ films increase with varying degrees after being annealed at different temperatures. It was reported that the refractive index is closely related to the density of materials, being lower at lower density. Consequently, the increase in the refractive index is caused by the stress release and densification during the annealing process [38, 39]. Furthermore, for the 20 nm La_2_O_3_ film, an abrupt increase in the refractive index could be observed when the annealing temperature increased from 400 to 600 °C, indicating an aggressive enhancement in the packing density upon crystallization. As a result, after being annealed at 600 °C, the 20 nm La_2_O_3_ film shows an index of refraction of 1.943 at the wavelength of 632.8 nm, which is much higher than that of the as-grown film (1.838). The refractive indexes obtained in this work are of good comparability with the results reported by Armelao et al. [1] and Kukli et al. [40].

**Fig. 5:**
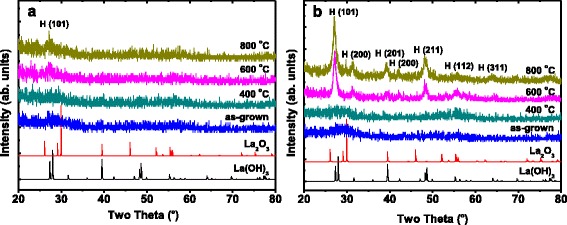
GIXRD diffractograms for as-grown and annealed La_2_O_3_ after being exposed to air for 48 h. **a** 10 nm La_2_O_3_ films annealed at 600 °C and **b** 20 nm La_2_O_3_ films annealed at 600 °C. Hexagonal La_2_O_3_ and hexagonal La(OH)_3_ patterns are added for comparison

**Fig. 6:**
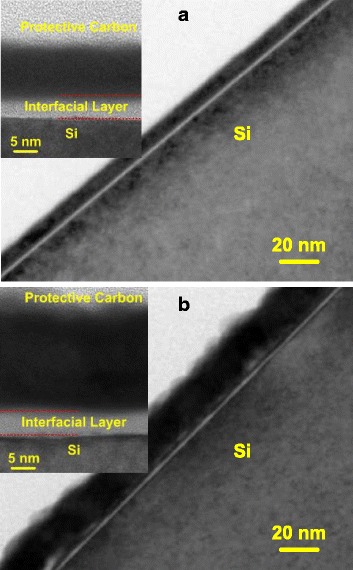
HRTEM images for the annealed La_2_O_3_ films after being exposed to air for 48 h. **a** 10 nm La_2_O_3_ film annealed at 600 °C and **b** 20 nm La_2_O_3_ film annealed at 600 °C

Figure 5 illustrates the GIXRD diffractograms for the as-grown and annealed La_2_O_3_ films after being exposed to air in clean room environment with a relative humidity of 50% for 48 h. Compared with the GIXRD diffractograms obtained before the air exposure as displayed in Fig. 1, almost all the GIXRD peaks attributed to *h*-La_2_O_3_ disappear, whereas new peaks attributed to *h*-La(OH)_3_ appear due to the hygroscopicity of La_2_O_3_ [22, 23, 41]. It is noteworthy that strong *h*-La(OH)_3_ phase peaks are only found in the well crystallized samples such as the 20 nm La_2_O_3_ films annealed at 600 and 800 °C, while few weak peaks are observed in the amorphous disordered and nanometer-sized crystallographic samples. Besides, it seems that the air exposure has a much heavier effect on the 20 nm La_2_O_3_ than that on the 10 nm La_2_O_3_. For clarity, cross-sectional HRTEM measurements on the annealed 10 and 20 nm La_2_O_3_ films after the air exposure were performed. The cross-sectional HRTEM image of the annealed 20 nm La_2_O_3_ after being exposed to air is shown in Fig. 6b, in which much more uneven interface and surface are observed than what can be found in the 10 nm La_2_O_3_. The deteriorations in the interface and surface properties are attributed to the degradation in the film density caused by the conversion from *h*-La_2_O_3_ to *h*-La(OH)_3_. With the time exposed to air, the amount of La(OH)_3_ in La_2_O_3_ film increases and then the density of the film is degraded, resulting in the changes of the surface and interfacial morphologies [42]. However, the existence of large mounts of LaSiO in the 10 nm La_2_O_3_ enhances the stability of the film structure, providing a high immunity against moisture ambient.

## Conclusions

The crystallization of La_2_O_3_ film grown by atomic layer deposition on Si substrate is restricted by the thickness of the film and the post-deposition annealing temperature. For thin (~10 nm) La_2_O_3_ film, only nanometer-sized crystals are formed after the annealing treatment due to the diffusion of Si substrate. For thick (~20 nm) La_2_O_3_, films can be mainly crystallized into *h*-La_2_O_3_ upon RTA performed in vacuum environment at 600 °C. After being crystallized, the refractive index of La_2_O_3_ film increases dramatically, while the bandgap is slightly decreased. After an exposure to air for 48 h, the *h*-La_2_O_3_ films are converted into *h*-La(OH)_3_ due to the hygroscopicity of La_2_O_3_.

## Abbreviations

ALD: Atomic layer deposition
EDX: Energy dispersive X-ray spectroscopy
GIXRD: Grazing incidence X-ray diffraction
HRTEM: High-resolution transmission electron microscopy
IL: Interfacial layer
RTA: Rapid thermal annealing
SE: Spectroscopic ellipsometry
XPS: X-ray photoelectron spectroscopy
